# Plasma Amino Acid Profile in Children with Autism Spectrum Disorder in Southern China: Analysis of 110 Cases

**DOI:** 10.1007/s10803-022-05829-z

**Published:** 2023-01-18

**Authors:** Wen-Xiong Chen, Yi-Ru Chen, Min-Zhi Peng, Xian Liu, Yan-Na Cai, Zhi-Fang Huang, Si-Yuan Yang, Jing-Yu Huang, Ruo-Han Wang, Peng Yi, Li Liu

**Affiliations:** 1 Department of Neurology, Guangzhou Women and Children’s Medical Center, Guangzhou Medical University, Guangzhou, China; 2 The Assessment and Intervention Center for Autistic Children, Guangzhou Women and Children’s Medical Center, Guangzhou Medical University, Guangzhou, China; 3 Department of Genetics and Endocrinology, Guangzhou Women and Children’s Medical Center, Guangzhou Medical University, Guangzhou, China; 4 Division of Birth Cohort Study, Guangzhou Women and Children’s Medical Center, Guangzhou Medical University, Guangzhou, China

**Keywords:** Autism spectrum disorder, Amino acid, Plasma, Clinical phenotypes, Severity of autism, Children

## Abstract

To retrospectively explore the characteristics of plasma amino acids (PAAs) in children with autism spectrum disorder and their clinical association via case-control study. A total of 110 autistic and 55 healthy children were recruited from 2014 to 2018. The clinical phenotypes included severity of autism, cognition, adaptability, and regression. Compared with the control group, autistic children had significantly elevated glutamate, γ-Amino-n-butyric acid, glutamine, sarcosine, δ-aminolevulinic acid, glycine and citrulline. In contrast, their plasma level of ethanolamine, phenylalanine, tryptophan, homocysteine, pyroglutamic acid, hydroxyproline, ornithine, histidine, lysine, and glutathione were significantly lower. Elevated neuroactive amino acids (glutamate) and decreased essential amino acids were mostly distinct characteristics of PAAs of autistic children. Increased level of tryptophan might be associated with severity of autism.

## Introduction

Autism spectrum disorder (ASD) is a neurodevelopmental disorder characterized by impairments in social interaction and communication, repetitive behaviors and/or restricted interests(Association, 2013). The overall prevalence of ASD has been consistently increasing in recent years. The early study on autism conducted in the 1960s reported that its prevalence was about 4/10,000(Lotter, 1966), whereas the prevalence of ASD had significantly risen from 13.4 to 1000 children in 2010(CDC, 2014) to 27.9 in 2016 in the USA(Xu et al., 2019). A recent meta-analysis indicated that the prevalence of ASD in China was 26.5/10,000(Liu et al., 2018), although this was significantly lower than the reported prevalence abroad. In 2020, the prevalence of ASD among children aged 6–12 years was about 0.7% in China(Zhou et al., 2020).

Children with ASD might be disabled and required life-long care(Cheuk et al., 2011), which burdens patients, families, and the public-health system. Although the pathogenesis of ASD is still not clear, it has been widely recognized that ASD occurs due to a combination of genetic and environmental factors, with the former being predominant (Bai et al., 2019). However, it has been found that maternal folic acid supplementation strategies, such as intake timing and intake dosage, may aid in reducing in the risk of ASD in offspring(Liu, Zou, Sun, Wu, & Chen, 2021).

In recent years, the metabolic abnormalities in ASD have attracted increasing attention, especially abnormalities related to amino acids. Amino acids are organic compounds containing amino and carboxyl groups, which are the basic protein units that have an essential role in regulating the immune system, cell signaling and metabolism, neurotransmission and so on(Vargason et al., 2018). Although previous studies have found the differences in plasma amino acid levels between autistic children and healthy children(Aldred, Moore, Fitzgerald, & Waring, 2003; D’Eufemia et al., 1995; Hoshino et al., 1984), there are still some disagreements over abnormal plasma amino acid levels between autistic children and healthy children. These inconsistent results in plasma amino acid levels between autistic children and healthy children might be due to the following reasons: firstly, most previous studies were small sample size studies, besides a few relatively large sample sizes in case-control studies (i.e., the sample size in ASD group ≥ 50 cases)(Adams et al., 2011; Cai, Ding, Zhang, Xue, & Wang, 2016; Naushad, Jain, Prasad, Naik, & Akella, 2013; Vargason et al., 2018; Xing, Lv, You, Zou, & Deng, 2021; Zou et al., 2020), among which, only one study had ASD group ≥ 100 cases(Naushad et al., 2013). Secondly, some studies did not match ASD and the control group for sex and age (Shimmura et al., 2011). Children of different age and sex have different levels of amino acids (Lepage, McDonald, Dallaire, & Lambert, 1997; Proenza, Crespí, Roca, & Palou, 2001); therefore it is important to match age and sex in the case-control study. Thirdly, some participants did not fast before a collecting blood sample. Amino acids are metabolites in the human body and essential amino acids are obtained from food, different diets affect the content of amino acids, and consequently, influencing the accuracy of plasma amino acid levels if no fasted was implemented before a collecting blood sample. Besides, vitamin deficiencies could be a risk factor of for ASD before analyzing the data. some studies did not exclude congenital vitamin B12 deficiency(Aldred et al., 2003). Although most studies had excluded congenital genetic metabolic diseases, acquired vitamin B12 deficiency was not excluded. Vitamin B12, a water-soluble vitamin that participates in the metabolism of methionine and homocysteine through the methionine cycle, has been given a lot of attention due to its involvement in the methylation process and folate cycle(Froese, Fowler, & Baumgartner, 2019). Also, a disorder of the methylation process or folate cycle has been proved to be related to embryonic development and neurodevelopment. Moreover, large cohort studies of plasma amino acids are still lacking in China, especially the lack of the studies regarding the relationship between amino acids levels and clinical phenotypes in ASD.

The aim of this large case-control retrospective study was to explore the characteristics of plasma amino acids (PAAs) in autistic children and their possible clinical phenotypic correlation, which might help with early identification and early intervention of ASD.

## Materials and methods

### Subjects

The autistic children were recruited, from the neuropsychological clinics specializing in ASD, Guangzhou Women and Children’s Medical Center, a national children’s medical center for the south central region between 2014 and 2018. The clinical history, comprehensive physical/neurological examination and relevant assessments were performed during the initial interview. The children who regularly participated in the normal physical examination at Guangzhou Women and Children’s Medical Center were recruited between 2014 and 2018 as a control group.

### Inclusion Criteria in ASD Group and Control Group

Children who met the following inclusion criteria were diagnosed with ASD by two independent specialist clinicians:(1) Diagnostic and Statistical Manual of Mental Disorder, Fifth Edition (DSM-5); (2) Autism Diagnostic Interview-Revised (AID-R); (3) Autism Diagnostic Observation Schedule (ADOS); (4) 1–14 years old. And those who were diagnosed at the age of < 2 years old were also included (those kids were followed up, and a definitive diagnosis was performed when they reached at least two years old).

The healthy children matched for age and sex were recruited as a control group. The healthy children in the control group were included if they met the following criteria: (1) with no diagnostic criteria for ASD; (2) regularly visited the outpatient of Guangzhou Women and Children’s Medical Center during 2014–2018 period; (3)1–14 years old.

### Exclusion Criteria in ASD Group and Control Group

Exclusion criteria for the ASD group: (1) children with hereditary/innate neurological and metabolic diseases; (2) children with acquired vitamin B12 deficiency and a history of vitamin supplement intake; (3) children with abnormal hepatic or renal function.

Exclusion criteria for the control group: in addition to meeting the exclusion criteria for the ASD group, other neurological/neurodevelopmental disorders were also excluded, such as ASD.

### Clinical Assessment in ASD Group

#### Methods for Assessment of the Severity of Autism

The Childhood Autism Rating Scale (CARS) was used to assess the severity of autism in ASD group. The CARS consisted of 15 items, where each item was scored on a scale of 1 to 4. The scores for the single items were summed together for a total score, according to which children were classified as not autistic (below 30), mild or moderately autistic (30–36.5), or severely autistic (above 36.5).

#### Methods for Assessment of Cognitive Ability

The Gesell Development Diagnosis Scale (GDDS) for development quotient (DQ), Wechsler Preschool and Primary Scale of Intelligence-IV (WPPSI-IV) or Wechsler Intelligence Scale for Children–IV (WISC-IV) for the intelligence quotient (IQ), were adopted for autistic children. According to the results of DQ/IQ, autistic children were divided into the high function group (HFG) (DQ/IQ ≥ 70) and the low function group (LFG) (DQ/IQ < 70).

#### Methods for Assessment of Regression

Autistic children were divided into regressive autism group and non-regressive autism group according to whether they had a regression. Regression was judged based on the children’s clinical symptoms and whether there was a loss of previously acquired skills, such as language/social communication regression(Barger, Campbell, & McDonough, 2013).

#### Methods for Assessment of Adaptive Function

The adaptive function was evaluated by the Modified Social Adaptation Scale for Infants-Junior Middle School Students(Jianduan et al., 2009; Zuo, 2016).

### Sample Collection and Testing

#### Sample Collection

Blood samples were collected from all children after fasting for 5–10 h. This study was approved by the Ethics Committee of Guangzhou Women and Children’s Medical Center. Written and signed consents were obtained from patients’ parents or guardians.

#### Plasma Amino Acids Testing

The levels of plasma amino acids were measured by liquid chromatography-tandem mass spectrometry, which could quantify 48 amino acids (Peng et al., 2019). The test items included the following forty-eight amino acids: Ethanolamine, Pyroglutamic acid, Glycine, Alanine, β-​Alanine, Sarcosine, α-​Amino-​n-​butyric acid, γ-​Amino-​n-​butyric acid, β-​Aminoisobutyric acid, Serine, Proline, Valine, Threonine, Pipecolic acid, δ-Aminolevulinic acid, N-acetyl-aspartic acid, Hydroxyproline, Leucine, Isoleucine, N-glycyl-glycine, Glutamine, Methionine, 3-Methyl-Histine, 1-Methyl-Histine, Phenylalanine, N-glycyl-proline, Citrulline, Aspartic acid, Glutamic acid, Arginine, α-​Aminoadipic acid, Tryptophan, Homocysteine, Cysteinine, Ornithine, Histidine, Lysine, Tyrosine, Saccharopine, Cystathionine, Glutathione, Kynurenine, Asparagine,Homoarginine, Homocitrulline, 5-Hydroxylysine,γ-Carboxy-glutamic acid, and Argininosuccinic acid. The concentration of plasma methylmalonic acid was determined by the stable-isotope dilution-liquid chromatography tandem mass spectrometry method according to the published protocol(Magera, Helgeson, Matern, & Rinaldo, 2000) .

### Statistical Analysis

All statistical analyses were carried out using IBMSPSS17.0 software. Firstly, the differences between PAAs in the ASD group and control group were tested by using a nonparametric Mann-Whitney Rank Sum Test. Secondly, the correlation between PAAs and the adaptability, cognition, severity of autism, and regression was assessed using logistic regression analysis respectively in the ASD group. P value < 0.05 (two-sided) was considered to be statistically significant.

## Results

### Demographic Data

#### Comparison of Demographic Data Between ASD and Control Group

A total of 165 Chinese children, 110 autistic children and 55 healthy control children matched for age and sex were included in the present study. Table 1 presents the distribution of age and gender between the ASD and the control group. There were no significant differences in age/sex between groups (P>0.05). Also, there were no significant differences in terms of the level of plasma methylmalonic acid between the ASD and control group (P > 0.05) (Data not shown).

**Table 1:** Comparison of demographic data between ASD and control group

Variables	ASD Mean ± SD	Control Mean ± SD	P-values
**Age** (year)	3.22 ± 1.18	3.37 ± 1.23	0.434
**Sex**	n(%)	n(%)	0.446
Female	15(13.6%)	10(18.2%)	
Male	95(86.4%)	45(81.8%)	

ASD: Autism Spectrum Disorder;SD: Standard deviation

#### Comparison of Demographic Data in ASD Group

In the ASD group, 96 (87.27%) autistic children were assessed for DQ/IQ. Among them, there were 18 autistic children in the high function group (HFG) (DQ/IQ ≥ 70), accounting for 18.75%. There were no significant differences in demographic data between HFG and LFG group (P > 0.05)**(**Table 2**)**.

**Table 2:** Comparison of demographic data in ASD group

Group	Age(year)	P-values	Sex(n(%))		P-values
			Female	Male	
**Cognition**		0.835			0.669
HFG	3.11 ± 1.34		3(16.67%)	15(83.33%)	
LFG	3.17 ± 1.08		10(12.82%)	68(72.92%)	
**Severity of autism**		0.588			0.557
Severe	3.10 ± 1.25		4(17.39%)	19(82.61%)	
Mild to moderate	3.25 ± 1.17		11(12.94%)	76(87.36%)	
**Regression**		0.281			0.354
Regression	2.92 ± 1.06		1(6.25%)	15(93.75%)	
Non- regression	3.26 ± 1.19		14(14.89%)	80(85.11%)	
**Adaptability**		0.760			0.258
Normal	3.02 ± 1.31		3(33.33%)	6(66.67%)	
Mild	3.08 ± 0.75		4(18.18%)	18(81.82%)	
Edge	2.97 ± 0.91		4(9.76%)	37(90.24%)	
Moderate	3.51 ± 1.38		1(5.56%)	17(94.44%)	
Severe	6.17 ± 2.95		0(0%)	2(100%)	

ASD: Autism Spectrum Disorder; DQ: Developmental quotient; HFG: High functional group (DQ/IQ ≥ 70); IQ: Intellectual disability; LFG: Low functional group (DQ/IQ < 70).

Regarding the severity of autism, there were 22 (20%) children with severe autism, whereas the remaining 88 (80%) were with mild to moderate autism. There were no significant differences in demographic data between groups with different severity of autism (P > 0.05) **(**Table 2**).**

In terms of regression, 16 (14.5%) autistic children had regression, whereas the remaining 94 (85.5%) had no regression. There were no significant differences in the demographic data between the regressive autism group and the non-regressive autism group (P > 0.05) **(**Table 2**)**.

Considering adaptability of 110 autistic children, there were no significant differences in demographic data between both groups in view of the different domains of adaptability (P > 0.05) **(**Table 2**)**.

#### Comparison of the Differences of Plasma Amino Acids Between ASD and Control Group

A total of 43 amino acids were finally analyzed as the five amino acids including homoarginine, homocitrulline, 5-hydroxylysine,γ-carboxy-glutamic acid, and argininosuccinic acid, were excluded due to the detection limit < 0.01µmol/L. Among those finally analyzed amino acids, shown in Table 3, there were significant differences in the plasma levels of 17 amino acids between ASD and the control group. Specifically, compared with the control group, autistic children showed significantly elevated plasma level of glutamic acid, γ-​amino-​n-​butyric acid, glutamine, sarcosine, δ-aminolevulinic acid, glycine, and citrulline, and significantly decreased plasma level of ethanolamine, phenylalanine, tryptophan, homocysteine, pyroglutamic acid, hydroxyproline, ornithine, histidine, lysine, and glutathione (all P > 0.05).

**Table 3:** Comparisons the differences of plasma amino acids between ASD and control group

Amino acid (µmol/L)	ASD group	Control group	Z-values	P-values
Ethanolamine	71.75	105.49	-4.27	<0.001
Pyroglutamic acid	23.55	51.39	-4.97	<0.001
Glycine	90.24	68.53	2.75	0.006
Sarcosine	104.15	40.69	8.04	<0.001
γ-​Amino-​n-​butyric acid	97.84	53.32	5.65	<0.001
δ-Aminolevulinic acid	90.62	55.60	4.62	<0.001
Hydroxyproline	75.09	98.82	-3.01	0.003
Glutamine	95.40	58.21	4.71	<0.001
Phenylalanine	76.90	95.20	-2.32	0.020
Citrulline	88.54	71.93	2.11	0.035
Glutamate	100.90	47.20	6.81	<0.001
Tryptophan	75.19	98.62	-2.97	0.003
Homocysteine	67.62	113.76	-5.85	<0.001
Ornithine	72.68	103.64	-3.92	<0.001
Histidine	77.12	94.75	-2.24	0.025
Lysine	76.17	99.66	-2.60	0.009
Glutathione	74.90	99.20	-3.08	0.002

ASD: Autism Spectrum Disorder

### The Association Between Plasma Amino Acid Levels and Clinical Phenotype in ASD Group

As shown in Table 4, we further analyzed the correlation between plasma amino acid levels and clinical symptoms of autism in the ASD group, and analyzed the clinical symptoms, including adaptability, cognition, severity of autism, and regression.

**Table 4:** The correlation between plasma amino acid and clinical phenotype in ASD group

Amino acid(µmol/L)	Crude OR(95%Cl)		Adjusted OR(95%Cl)*
Severity of autism			
Tryptophan	1.034(1.002–1.067)		1.033(1.001–1.066)
Regression			
Glutathione	1.241(0.994–1.550)	1.252(0.999–1.571)	

ASD: Autism Spectrum Disorder

*adjusted sex and age

Regarding the severity of autism, a significant correlation was found with the plasma level of tryptophan (P < 0.05) (Crude OR, 1.034;95%CI:1.002–1.067)**(**Table 4**)**; the higher the plasma level of tryptophan level, the worse the severity of autism in autistic children. After adjusting sex and age, the trend remained (Adjusted OR:1.033; 95%OR:1.001–1.066).

The level of glutathione was also positive with the regression of autistic children, but without significance (Crude OR,1.241;95%CI:0.994–1.550)(Table 4).

However, there was no significant difference between the level of PAAs and respective clinical phenotypes including cognition, or adaptability.

## Discussion

In the current study, we found that the elevated plasma levels of neuroactive amino acids (glutamate) and decreased plasma levels of essential amino acids (lysine, tryptophan, phenylalanine, histidine) represented mostly distinct characteristics of plasma amino acids in autistic children. Also to the best of our knowledge, this is the first study that analyzed the correlation between PPAs and a set of clinical phenotypes, including adaptability, cognition ability, severity of autism and regression in ASD.

Recently, there has been a growing interest in the differences in amino acids between autistic and healthy children. However, the outcomes from previously published studies remain debatable. Secondly, most previous studies did not explore the correlation between plasma amino acids and the clinical phenotype of autism. Moreover, besides a few relatively large sample sizes in case-control studies (i.e. sample size in ASD group ≥ 50 cases; defined in the current study)(Adams et al., 2011; Cai et al., 2016; Naushad et al., 2013; Vargason et al., 2018; Xing et al., 2021; Zou et al., 2020), most previous studies were small sample sizes, as is shown in Table 5, among which, only two studies(Naushad et al., 2013) (including current study) had sample sizes in the ASD group ≥ 100 cases.

**Table 5:** Comparison of case-control studies of plasma amino acids in children with ASD

Author (country/region; race; publication year; journal)	ASD group n(male/female;age(M ± SD)	Control group n(male/female;age(M ± SD)	Diagnostic criteria(ASD)	Types of Sample	Detection method	amino acids (AAs) outcomes		Cognition(DQ/IQ)	Severity of autism	Regression		Others	
1. Adams JB et al. (America/Arizona; Nil; 2011; Nutrition & Metabolism)	55(49/6; 10 ± 3.1y)	44(39/5; 11 ± 3.1y)	DSM-IV	Plasma	HPLC-MS/MS	Forty one plasma AAs measured. 1. ASD group: 1) Increased level: glutamate, serine, beta-amino isobutyrate, and homocysteine. 2) Decreased level: tryptophan, isoleucine, phenylalanine, tyrosine, and taurine. 2. Severity of autism: The proline, serine, ethanolamine, or beta-amino-isobutyrate correlated with the severity of autism for all three scales (PDD-BI/ATEC/SAS).		Nil	PDD-BI/ATEC/SAS	Nil		1. Fasting for 8-12 h. 2. Sex-age matched. 3. Children’ age 5–16 years old in both groups. 4. No usage of a vitamin/mineral supplement in the last 2 Mo. 5. No current use of any chelation treatment. 6. 20 out of 41 plasma AAs below the detection limit.	
2. Naushad SM et al. (India/Hyderabad; Nil;2013; Indian Journal of Biochemistry & Biophysis)	138(120/18; 4.4 ± 1.7y)	138(120/18; 4.4 ± 1.6y)	DSM-IV/ABC	Plasma	Reverse-phase HPLC	Ten plasma AAs tested. ASD group: 1) Increased level: glutamate, and asparagine. 2) Decreased: tryptophan, histidine, methionine, and phenylalanine.		DQ (ASD group: 30 < DQ < 80/control group: DQ > 80)	Nil	Nil		1. No special diet during the time of analysis. 2. Fasting blood samples. 3. ABC score: ASD group >68 score; Control group<21score. 4. Sex-age matched.	
3. Cai J et al. (China /Shangdong; Han; 2016;NeuroReport)	51(42/9; average age:3.69y)	Healthy control: 51(42/9; average age :3.69y); ID group: 51(42/9; average age :3.69y)	DSM-IV	Plasma	LC-MS/MS	Only detected glutamate. 1. ASD group: Increased level: glutamate. 2. Severity of autism: The increased level of glutamate positively associated with severity of autism.		Average FSIQ (ASD /control/ID group): 72.6/102 /68.7	Average CARS score (ASD/control/ID group): 41.1/21.8 /22.5	Nil		1. Medication free for at least 5 weeks. 2. BMI (ASD/Control/ID group):16.3/17.2/17 Kg/m^2^.	
4. Vargason T et al. (America/Arizona: Caucasian; 2018: Research Autism Spectrum Disorder)	64 (52/12; 11.8 ± 8.5y)	49 (40/9; 12.2 ± 7.6y)	ADOS/CARS	Plasma	GC/MS	Twenty two plasma AAs and related amino acid metabolites were measured. ASD group: Increased level: glutamate, serine and hydroxyproline.		RIAS: assess the IQ of ASD group children	CARS	Nil		1. Fasting overnight. 2. Age-sex matched. 3. ASD group: (45 children, 13 teens, 6 adults); Control group: (34 children, 10 teens, 5 adults). 4. 20 out of plasma AAs excluded for final analysis because of below the detection limit.	
5. Zou M et al. (China/Harbin; Han; 2020;Neurotox Research)	70 (62/8; 5.44 ± 1.36y)	70 (62/8; 5.12 ± 0.79y)	DSM-5	Plasma	LC-MS/MS	Twenty one plasma AAs measured. 1. ASD group: Increased level: Arginine, cysteine, homocysteine, histidine, methionine, serine, tyrosine and valine. 2. Severity of autism: The level of homocysteine was positively correlated with the severity of autism.		Nil	ADOS-CSS	Nil		1. Standardized diet and moderate physical activity for one week. 2. Fasting blood samples.	
6. Xing Y et al. (China /Guangzhou; Han; 2021; Psychiatry Research)	60(49/11; 42.86 ± 11.03Mo)	30(25/5; 39.32 ± 12.88Mo)	DSM-IV	Serum	TMS	Twenty two serum AAs measured. 1. ASD group: 1) Increased level: asparagine. 2) Decreased level: lysine, tryptophan, phenylalanine, methionine, leucine, valine, threonine, glutamine, glycine, alanine, citrulline, cysteine, serine, tyrosine acid, and proline. 2. The serum glutamate/glutamine (Glu/Gln) ratio was elevated in the ASD PIQ ≥ 70 group.		ASD group: 15 out of 60 children (3y and11Mo): assessed with C-WYCSI(FIQ: 48.0 ± 18.7; VIQ: 46.4 ± 12.9; PIQ: 58.8 ± 22.4).	Nil	Nil		1. BMI for each child. 2. Fast for 10 h.	
7. Current research	110(95/15; 3.22 ± 1.18y)	55(45/10; 3.37 ± 1.23y)	DSM-5/ADI-R/ADOS	Plasma	LC-MS/MS		Forty eight plasma AAs measured and 43 plasma AAs analyzed. 1. ASD group: 1) Elevated level: glutamate, γ-Amino-n-butyric acid, glutamine, sarcosine, δ-Aminolevulinic acid, glycine and citrulline. 2) Decreased level: ethanolamine, phenylalanine, tryptophan, homocysteine, pyroglutamic acid, hydroxyproline, ornithine, histidine, lysine, and glutathione. 2. Severity of autism: The level of tryptophan distinct lower in severe group than in mild and moderate group.	DQ/IQ: by GDDS/WISC-I V/WPPSI-IV): HFG: DQ/IQ ≥ 70 ;LFG: (DQ/IQ < 70)	CARS: Severe autism: CARS score ≥ 36.5; mild and moderate autism: 30 ≤ CARS socre < 36.5		ASD group: Regressive group and Non-regressive group		1. Fasting for 5-10 h. 2. Excluded vitamin B12 deficiency. 3. Age-sex matched. 4. 5 out of forty eight plasma AAs excluded due to below the detection limit.

ABC: Autism Behavior Scale; ADI-R: Autism Diagnostic Interview Revised; ADOS: Autism Diagnostic Observation Scale; ASD: Autism Spectrum Disorder; ATEC : Autism Evaluation Treatment Checklist; BMI: Body Mass Index; CARS: Childhood Autism Rating Scale; CSS: Calibration Severity Score; C-WYCSI: Chinese-Wechsler Young Children Scale of Intelligence; DQ: Development Quotient; DSM-IV: Diagnostic and Statistical Manual of Mental Disorders-IV; DSM-5: Diagnostic and Statistical Manual of Mental Disorders-5; FSIQ: Full Scale Intelligence Quotient; GC/MS: Gas Chromatography/Mass Spectrometry; GDDS: Gesell Development Diagnosis Scale; h: hour; HFG: High Functional Group; HPLC: High Performance Liquid Chromatography; ID: Intellectual Disability; LC-MS/MS: Liquid Chromatography-Tandem Mass Spectrometry; LFG: Low Functional Group; M: Mean; Mo: Month; PDD-BI: Pervasive Development Disorder-Behavior Inventory; PIQ: Performance Intelligence Quotient; RIAS: Reynolds Intelligence Assessment Scale; SAS: Severity of Autism Scale; SD: Standard Deviation; TMS:: Tandem Mass Spectrometry; VIQ: Verbal Intelligence Quotient; WISC-IV: Wechsler Intelligence Scale for Children-IV; WPPSI-IV: Wechsler Preschool and Primary Scale of Intelligence-IV; y: year

### The Differences Between ASD Children and Healthy Children: Plasma Amino Acids

The present study found that Compared to healthy children, autistic children had elevated glutamate, glutamine, γ-aminobutyric acid, glycine and citrulline, being consistent with previous studies(Adams et al., 2011; Cai et al., 2016; Naushad et al., 2013), and reduced lysine, tryptophan, phenylalanine and histidine, which is in line with previous researches(Adams et al., 2011; Naushad et al., 2013; Ormstad et al., 2018; Xing et al., 2021) **(**Table 3**)**. Glutamate is an important excitatory neurotransmitter in the brain, and when elevated, it might be involved in the pathogenesis of ASD due to the following reasons: firstly, excessive glutamate leads to brain excitotoxicity by over-stimulating glutamate receptors in autism, causing neuronal oxidative stress and mitochondrial damage(Blaylock & Strunecka, 2009). Secondly, it has an important role in children’s early cerebral cortex development. Excessive glutamate in autism disrupts the balance in glutamate metabolism, causing abnormal development in the cerebral cortex(Manent & Represa, 2007). Moreover, excessive glutamate would cause neuronal cell membrane, cytoskeleton and DNA damage by activating a series of enzymes involved in the development and function of normal neurons(Choi, 1985). Among these activated enzymes, some would further increase the glutamate level and cause severe excitotoxicity by changing the permeability of the blood-brain barrier(Melendez, Melathe, Rodriguez, Mazurkiewicz, & Davies, 1999). Besides, as glutamine is the amide of glutamate, excessive glutamate would cause glutamine abnormalities(Yüksel & Öngür, 2010).

In contrast, glycine and γ-aminobutyric acid are inhibitory neurotransmitters that have an important role in cell proliferation, differentiation and synaptic maturation in the central nervous system(Ito, 2016; Owens & Kriegstein, 2002). Increased γ-aminobutyric acid and glycine in autistic children can disturb the excitation/inhibition balance in brain, which might lead to autism(Marotta et al., 2020; Zheng, Wang, Li, Rauw, & Baker, 2017).

Essential amino acids in the human body must be supplied by food; however, some autistic children may have eating difficulties and gastrointestinal symptoms, and be very picky about the taste and color of food(Kral, Eriksen, Souders, & Pinto-Martin, 2013). Accordingly, decreased essential amino acids (lysine, tryptophan, phenylalanine, histidine) in autistic children might be partially due to insufficient food intake or poor eating habits. Besides, we also found that the plasma levels of ethanolamine and glutathione (reduced) in the ASD group were decreased, which is consistent with the previous studies(Bala et al., 2016; Geier et al., 2009; James et al., 2004). Ethanolamine is involved in synthesizing phosphatidylethanolamine, and reduced ethanolamine in autistic children might cause chronic oxidative stress via decreased phosphatidylethanolamine synthesis(Wang et al., 2014). Glutathione is a tri-peptide involved in the redox balance of glutathione in the intracellular environment. The intracellular environment is maintained by a high glutathione (reduced)/glutathione (oxidative) ratio(Schafer & Buettner, 2001), which regulates a wide range of cell functions, including the scavenging of oxygen free radicals, cell membrane integrity, signal transduction, and so on(Dickinson et al., 2003). Therefore, reduced glutathione in autistic children may disrupt the redox balance of glutathione, thus further aggravating oxidative stress.

Interestingly, we also found that the plasma levels of sarcosine and δ-aminolevulinic acid were elevated. Sarcosine is an intermediate product of glycine metabolism, and the increased sarcosine level in our study might be due to increased glycine level, although Adams et al (Adams et al., 2011) found no significant difference in the sarcosine plasma level between the autistic children and neurotypical children in their study. However, most measurements of secondary plasmas amino acids and amino acid metabolites, including sarcosine being below the detection limit of 0.05 umoles/100ml and the large range age span of recruited subjects (5-16y) limited the interpretation of the outcomes of their study(Adams et al., 2011). Vargason et al(Vargason et al., 2018) also measured the level of Sarcosine, but omitted it from further analysis due to the subjects’ intervention issue. In addition, besides children, they(Vargason et al., 2018) also recruited adult subjects, and therefore, the age span of included subjects was large (11.8 ± 8.5y), which made it difficult to compare their PPA’s outcomes with current study and other studies that only included children’ subjects, as the level of plasma amino acids substantially varies with age(Lepage et al., 1997).

The plasma level of δ-aminolevulinic acid or pyroglutamic acid was not reported in the previous large sample sizes case-control studies **(**Table 5**)**. In vivo, δ-aminolevulinic acid as the precursor of heme, is produced by glycine and succinyl-CoA under the δ-amino-γ-levulinic acid (ALA) synthetase(McLeod, Mack, & Brown, 1991), and δ-aminolevulinic acid must activate mitochondria so that it can convert into heme in the cell(Malik & Djaldetti, 1979). Most studies have indicated mitochondrial dysfunction and oxidative stress as the neuropathological basis of autism (Gorman et al., 2015; Rossignol & Frye, 2012). Therefore, the elevated δ-aminolevulinic acid in autistic children might cause mitochondrial dysfunction and oxidative stress. However, a recent animal model study showed that δ-aminolevulinic acid could inhibit oxidative stress and ameliorate autistic-like behaviors for the prenatal valproic acid-exposed rats(Matsuo, Yabuki, & Fukunaga, 2020), which implicate that accumulation of δ-aminolevulinic acid may result from autism-induced mitochondria dysfunction. The further studies needed regarding the relationship between δ-aminolevulinic acid and pathogenesis of autism.

Pyroglutamic acid is cyclized to form lactams from free amino groups of glutamate or glutamine. Pyroglutamic acid can antagonize nerve excitement by inhibiting glutamate(Abraham & Podell, 1981).The reduced plasma level of pyroglutamic acid in autistic children revealed in our study might further aggravate the neuroexcitatory toxicity of autism by reducing the inhibitory effect of pyroglutamate acid on glutamate.

In addition, we found that the levels of homocysteine, hydroxyproline and ornithine in autism were reduced, which were contrary to previous researches’ results (Vargason et al., 2018; Zou et al., 2020). The homocysteine is a non-protein sulfurized amino acid, which might vary with different age and sex(Guo, Li, & Ding, 2020). Also, congenital hyperhomocysteinemia may occur due to a lack of cofactors such as vitamin B6, vitamin B12, and folic acid(Bhatia & Singh, 2015). Most previous studies did not exclude children with acquired vitamin B12 deficiency, so homocysteine might be increased in their studies. Moreover, previous study has shown that abnormal transsulfur metabolism might be involved in ASD(James et al., 2006). Therefore, the reduced plasma of homocysteine in our study might lead to the dysfunction of transsulfur metabolism, contributing to the occurrence of ASD by its metabolic pathway (transsulfuration pathway). Hydroxyproline is a component of collagen, which is the basis for all connective tissues (tendon, bones and cartilage) (Li & Wu, 2018). Previous research has shown that the increased hydroxyproline levels might be associated with joint hypermobility in autistic children (Bala et al., 2016). These children also manifested repetitive behaviors, such as clapping, waving, etc.

### The Correlation Between Plasma Amino Acid and Clinical Phenotype

The correlation between plasma amino acid and clinical phenotype was further analyzed using logistic regression analysis in the autistic children group.

In terms of severity of autism, there were positively correlation between severity of autism and the plasma level of tryptophan (Table 4); the higher the plasma tryptophan level, the worse the severity of autism.

The plasma level of tryptophan was reduced in the current study, which was in line with previous studies(Adams et al., 2011; Naushad et al., 2013; Xing et al., 2021). Autistic children often suffer from picky eating and intestinal disorders, which might result in decreased protein intake, and/or insufficient digestion and absorption of protein into amino acids. Tryptophan is an essential amino acid in the human body, which is taken from outside to meet the body’s needs. Autistic children tend to have poor eating behaviors and digestive dysfunction, which may partially lead to the decreased plasma level of tryptophan (Xing et al., 2021). Tryptophan is a precursor of important compounds, such as serotonin and quinolinic acid, which are involved in neurodevelopment and synaptogenesis(Boccuto et al., 2013). A deficiency in tryptophan, which caused the decreased synaptic serotonin, resulted in the worsened repetitive behaviors and irritability in autism(C. McDougle et al., 1993). Decreased blood tryptophan levels of autistic children have been found in several studies(Adams et al., 2011; C. McDougle et al., 1993; Naushad et al., 2013; Xing et al., 2021), but not reported in all studies(Muller, Anacker, & Veenstra-VanderWeele, 2016). A meta-analysis(Gabriele, Sacco, & Persico, 2014) revealed that blood serotonin level was increased in some autistic children, although most included studies in the meta-analysis being small sample sizes studies. A large sample size studies regarding the relationship between the levels of tryptophan and serotonin in the blood of autistic children are needed.

Besides, tryptophan is a precursor of quinolinic acid, and the kynurenine pathway (KP) is the primary route for tryptophan catabolism in the liver(Davis & Liu, 2015). The KP of tryptophan degradation is activated in neuroinflammatory states(Lim et al., 2016), creating KP metabolites kynurenic acid and quinolinic acid. Different studies have reported different findings with reference to the results of KP metabolites. Lim et al. (Lim et al., 2016) reported that autistic children has increased tryptophan, kynurenic acid and quinolinic acid, whereas Bryn et al. (Bryn, Verkerk, Skjeldal, Saugstad, & Ormstad, 2017) found that autistic children had decreased tryptophan, kynurenic acid and quinolinic acid. In the present study, we found that autistic children had decreased tryptophan and unchanged kynurenic acid. Inconsistent results might be due to different sample sizes of cases in the different studies. Secondly, quinolinic acid is the structural precursor of NAD+, which is a critical energy carrier in mitochondria(Stone & Darlington, 2002). Moreover, the low tryptophan plasma level in autistic children might cause mitochondrial dysfunction, which affect neuronal development and morphology, neurite overgrowth, and synaptic plasticity. What more, the KP and the gut microbiome tend to influence each other, which may further reduce tryptophan intake in autism(Van der Leek, Yanishevsky, & Kozyrskyj, 2017).

Otherwise, we further explore the association between tryptophan level and severity of autism. The interesting finding in our research is that the higher the tryptophan level, the more severity of autism. Different previous studies had different opinions on the relationship between tryptophan levels of blood and severity of symptoms of autistic children. Some studies(C. J. McDougle et al., 1996; Naushad et al., 2013) reported the decreased tryptophan levels would deteriorate the symptoms of autistic patients, whereas other studies(Bergwerff, Luman, Blom, & Oosterlaan, 2016; Jennings & Basiri, 2022; Kaluzna-Czaplinska, Jozwik-Pruska, Chirumbolo, & Bjorklund, 2017) found that the higher tryptophan the more severe symptoms of autistic patients. Previous study also found tryptophan levels were higher in the children with Asperger’s syndrome than in the control children (Ormstad et al. 2018), which was partly consistent with our study views. The elevated (5-HT + 5-HTP)/tryptophan ratio was found in Asperger’s syndrome, which lowered the activity of the peripheral 5-HT synthesis pathway and increased plasma tryptophan levels(Ormstad et al. 2018). This result gives us the reflection that there may exist a balance downstream of the tryptophan pathway. Our finding indicates that more severe group of autistic children might be accompanied by the lowered activity of the serotonin synthesis pathway, which might contribute to increase plasma tryptophan levels. It shows that the serotonergic pathophysiology may be more impaired in the more severe group of autistic children. Inadequate intake and impaired metabolic conversion of serotonin lead to more severe symptoms of autism. Regretfully, the study did not measure the downstream metabolites of the tryptophan pathway. Meanwhile, more details about the underlying mechanisms of kynurenine pathway metabolites should also be considered. This present study warrants the need for a comprehensive focus on the expression of upstream and downstream products of the tryptophan metabolic pathway and kynurenine pathway in the future.

Moreover, some previous studies also analyzed the correlation between plasma amino acids and the severity of autism(Adams et al., 2011; Cai et al., 2016; Zou et al., 2020). Adams et al. found the respective plasma level of ethanolamine, proline, serine or beta-amino-isobutyrate was correlated with the severity of autism(Adams et al., 2011), although the severity of autism was evaluated by the three assessment tools, i.e., Pervasive Development Disorder Behavior Inventory (PDD-BI), Autism Evaluation Treatment Checklist (ATEC), and Severity of Autism Scale (SAS) (Table 5) in their study. Ethanolamine, obtained from the diet, is involved in synthesizing phosphatidylethanolamine(Wang et al., 2014). The intake of ethanolamine differs with different ages, and a large age span (5-16y) of the subjects included in their study might impact interpretation of outcomes to some extent. β-aminoisobutyric acid is a nonproteinogenic amino acid, a known catabolite of thymine, and one of the four nucleobases in the nucleic acid of DNA(Tanianskii et al., 2019). Adams et al. showed that increased β-aminoisobutyric acid might increase the rate of DNA turnover and inhibit the conversion of β-aminoisobutyric acid to produce energy in the citric acid cycle of mitochondria(Adams et al., 2011). Although Adams et al. showed that the plasma level of proline and severity of autism were correlated, they did not found that any difference in proline level between the ASD and control groups in their study. Also, Adams et al. found that the plasma level of serine was increased in the ASD group, which was consistent with previous researches(Vargason et al., 2018; Zou et al., 2020), and they also found that the level of serine was correlated with the severity of autism. Serine is the main contributor of one-carbon units, and one-carbon metabolism is associated with redox metabolism and methylation(Vargason et al., 2018). The impairment in methylation and oxidative stress affect the occurrence of ASD. Increased serine might affect one-carbon metabolism, thus leading to ASD.

Cai et al.(Cai et al., 2016) found the plasma level of glutamate was positively associated with increasing severity of ASD. In their study, Cai and colleagues showed three possible mechanisms in their study, which were mostly related to the excitotoxicity of glutamate.

In 2020, Zou et al(Zou et al., 2020) also found that the level of homocysteine was positively correlated with the severity of autism, although they evaluated the severity of autistic children with ADOS-CSS (Table 5).

Regarding the regression, although there were no significant differences between plasma amino acids and regression in the current study, the level of glutathione might have a significant trend toward associate with regression (Table 4). Glutathione is a tripeptide, composed of glutamic acid, cysteine, and glycine, involved in the redox balance of glutathione in the intracellular environment. In addition, a previous study showed that the serum glutamate/glutamine ratio was elevated in ASD PIQ ≥ 70 group(Xing et al., 2021).

However, when the correlation was analyzed between plasma amino acids and other respective clinical phenotypes, including cognition and adaptability, there were no significant differences in the current study.

## Strength and Limitation

The present study was a relatively large-sample case-control study that recruiting more than 100 cases. We tried to exclude other confounding factors, such as acquired vitamin B12 deficiency, age and sex-matched between groups, to ensure the accuracy and reliability of the study. We also measured the level of amino acids by the advanced detection method of LC-MS/MS. Currently, we measured and analyzed the largest number of PAAs among case-control studies **(**Table 5**)**. In addition, we further analyzed the correlation between plasma amino acid levels and a set of the clinical phenotypes of autistic children.

However, the present study has the following limitations: (1) this was retrospective research, so prospective large sample cohort studies are needed to further verify the findings of this study in the future; (2) the PAAs level was greatly affected by eating, it might be more accurate to dynamically monitor the changes of amino acids. Also, no information was collected on the patient’s underlying nutritional status; (3) there were no other neurodevelopmental groups, such as the ID group included in the current study, influencing outcomes’ representatives.

## Conclusion

There were significant differences in seventeen amino acids between ASD and the control group; Elevated levels of neuroactive amino acids (glutamate) and decreased essential amino acids exhibited mostly distinct characteristics of plasma amino acid in autistic children. Increased level of tryptophan might be associated with the severity of autism.

## References

[CR1] Abraham, G. N., & Podell, D. N. (1981). Pyroglutamic acid. Non-metabolic formation, function in proteins and peptides, and characteristics of the enzymes effecting its removal. *Mol Cell Biochem, 38 Spec No*(Pt 1), 181–190. doi:10.1007/bf00235695 10.1007/BF00235695 6117006

[CR2] Adams JB, Audhya T, McDonough-Means S, Rubin RA, Quig D, Geis E, Lee W (2011). Nutritional and metabolic status of children with autism vs. neurotypical children, and the association with autism severity. Nutr Metab (Lond).

[CR3] Aldred S, Moore KM, Fitzgerald M, Waring RH (2003). Plasma amino acid levels in children with autism and their families. Journal Of Autism And Developmental Disorders.

[CR4] Association, A. P. (2013). *The Diagnostic and Statistical Manual of Mental Disorders. 5th ed.Washington DC: American Psychiatric Publishing Inc,2013*.

[CR5] Bai D, Yip BHK, Windham GC, Sourander A, Francis R, Yoffe R, Sandin S (2019). Association of genetic and environmental factors with autism in a 5-Country cohort. JAMA Psychiatry.

[CR6] Bala KA, Doğan M, Mutluer T, Kaba S, Aslan O, Balahoroğlu R, Kocaman S (2016). Plasma amino acid profile in autism spectrum disorder (ASD). European Review For Medical And Pharmacological Sciences.

[CR7] Barger BD, Campbell JM, McDonough JD (2013). Prevalence and onset of regression within autism spectrum disorders: a meta-analytic review. Journal Of Autism And Developmental Disorders.

[CR8] Bergwerff CE, Luman M, Blom HJ, Oosterlaan J (2016). No tryptophan, tyrosine and phenylalanine abnormalities in children with Attention-Deficit/Hyperactivity disorder. PLoS One.

[CR9] Bhatia P, Singh N (2015). Homocysteine excess: delineating the possible mechanism of neurotoxicity and depression. Fundamental & Clinical Pharmacology.

[CR10] Blaylock RL, Strunecka A (2009). Immune-glutamatergic dysfunction as a central mechanism of the autism spectrum disorders. Current Medicinal Chemistry.

[CR11] Boccuto L, Chen CF, Pittman AR, Skinner CD, McCartney HJ, Jones K, Schwartz CE (2013). Decreased tryptophan metabolism in patients with autism spectrum disorders. Mol Autism.

[CR12] Bryn V, Verkerk R, Skjeldal OH, Saugstad OD, Ormstad H (2017). Kynurenine Pathway in Autism Spectrum Disorders in Children. Neuropsychobiology.

[CR13] Cai J, Ding L, Zhang JS, Xue J, Wang LZ (2016). Elevated plasma levels of glutamate in children with autism spectrum disorders. Neuroreport.

[CR14] CDC, D. D. M. N. S. Y., & P., P. (2014). Prevalence of autism spectrum disorder among children aged 8 years - autism and developmental disabilities monitoring network, 11 sites, United States, 2010. *MMWR Surveill Summ, 63*(2), 1–21. doi:10.15585/mmwr.ss6706a1 24670961

[CR15] Cheuk, D. K., Wong, V., & Chen, W. X. (2011). Acupuncture for autism spectrum disorders (ASD). *Cochrane Database Syst Rev*(9), Cd007849. doi:10.1002/14651858.CD007849.pub2 10.1002/14651858.CD007849.pub2 PMC8939294 21901712

[CR16] Choi DW (1985). Glutamate neurotoxicity in cortical cell culture is calcium dependent. Neuroscience Letters.

[CR17] D’Eufemia P, Finocchiaro R, Celli M, Viozzi L, Monteleone D, Giardini O (1995). Low serum tryptophan to large neutral amino acids ratio in idiopathic infantile autism. Biomedicine & Pharmacotherapy.

[CR18] Davis I, Liu A (2015). What is the tryptophan kynurenine pathway and why is it important to neurotherapeutics?. Expert Review Of Neurotherapeutics.

[CR19] Dickinson DA, Moellering DR, Iles KE, Patel RP, Levonen AL, Wigley A, Forman HJ (2003). Cytoprotection against oxidative stress and the regulation of glutathione synthesis. Biological Chemistry.

[CR20] Froese DS, Fowler B, Baumgartner MR (2019). Vitamin B(12), folate, and the methionine remethylation cycle-biochemistry, pathways, and regulation. Journal Of Inherited Metabolic Disease.

[CR21] Gabriele S, Sacco R, Persico AM (2014). Blood serotonin levels in autism spectrum disorder: a systematic review and meta-analysis. European Neuropsychopharmacology.

[CR22] Geier DA, Kern JK, Garver CR, Adams JB, Audhya T, Geier MR (2009). A prospective study of transsulfuration biomarkers in autistic disorders. Neurochemical Research.

[CR23] Gorman GS, Schaefer AM, Ng Y, Gomez N, Blakely EL, Alston CL, McFarland R (2015). Prevalence of nuclear and mitochondrial DNA mutations related to adult mitochondrial disease. Annals Of Neurology.

[CR24] Guo BQ, Li HB, Ding SB (2020). Blood homocysteine levels in children with autism spectrum disorder: an updated systematic review and meta-analysis. Psychiatry Research.

[CR25] Hoshino Y, Yamamoto T, Kaneko M, Tachibana R, Watanabe M, Ono Y, Kumashiro H (1984). Blood serotonin and free tryptophan concentration in autistic children. Neuropsychobiology.

[CR26] Ito S (2016). GABA and glycine in the developing brain. The Journal Of Physiological Sciences: Jps.

[CR27] James SJ, Cutler P, Melnyk S, Jernigan S, Janak L, Gaylor DW, Neubrander JA (2004). Metabolic biomarkers of increased oxidative stress and impaired methylation capacity in children with autism. American Journal Of Clinical Nutrition.

[CR28] James SJ, Melnyk S, Jernigan S, Cleves MA, Halsted CH, Wong DH, Gaylor DW (2006). Metabolic endophenotype and related genotypes are associated with oxidative stress in children with autism. Am J Med Genet B Neuropsychiatr Genet.

[CR29] Jennings, L., & Basiri, R. (2022). Amino acids, B Vitamins, and Choline May independently and collaboratively influence the incidence and core symptoms of Autism Spectrum Disorder. *Nutrients*, *14*(14), doi:10.3390/nu14142896. 10.3390/nu14142896 PMC9318435 35889852

[CR30] Jianduan Z, Huishan W, Shuhua S, Xiaonan H, Guoyan L, Guangli L, Junxin S (2009). Reliability and validity of standardized chinese version of Urban Infant-Toddler Social and Emotional Assessment. Early Human Development.

[CR31] Kaluzna-Czaplinska J, Jozwik-Pruska J, Chirumbolo S, Bjorklund G (2017). Tryptophan status in autism spectrum disorder and the influence of supplementation on its level. Metabolic Brain Disease.

[CR32] Kral TVE, Eriksen WT, Souders MC, Pinto-Martin JA (2013). Eating Behaviors, Diet Quality, and gastrointestinal symptoms in Children with Autism Spectrum Disorders: a brief review. Journal of Pediatric Nursing.

[CR33] Lepage N, McDonald N, Dallaire L, Lambert M (1997). Age-specific distribution of plasma amino acid concentrations in a healthy pediatric population. Clinical Chemistry.

[CR34] Li P, Wu G (2018). Roles of dietary glycine, proline, and hydroxyproline in collagen synthesis and animal growth. Amino Acids.

[CR35] Lim CK, Essa MM, de Paula Martins R, Lovejoy DB, Bilgin AA, Waly MI, Guillemin GJ (2016). Altered kynurenine pathway metabolism in autism: implication for immune-induced glutamatergic activity. Autism Research.

[CR36] Liu X, Chen WX, Lin SF, Chan FF, Shen SY, Qiu X (2018). Prevalence of autism spectrum disorders among children in China:a systematic review and meta-analysis. Chinese Journal of Child Care.

[CR37] Liu X, Zou M, Sun C, Wu L, Chen WX (2021). Prenatal folic acid supplements and offspring’s Autism Spectrum disorder: a Meta-analysis and Meta-regression. Journal Of Autism And Developmental Disorders.

[CR38] Lotter V (1966). Epidemiology of autistic conditions in young children. Social Psychiatry.

[CR39] Magera MJ, Helgeson JK, Matern D, Rinaldo P (2000). Methylmalonic acid measured in plasma and urine by stable-isotope dilution and electrospray tandem mass spectrometry. Clinical Chemistry.

[CR40] Malik Z, Djaldetti M (1979). 5-Aminolevulinic acid stimulation of porphyrin and hemoglobin synthesis by uninduced friend erythroleukemic cells. Cell Differ.

[CR41] Manent JB, Represa A (2007). Neurotransmitters and brain maturation: early paracrine actions of GABA and glutamate modulate neuronal migration. The Neuroscientist : A Review Journal Bringing Neurobiology, Neurology And Psychiatry.

[CR42] Marotta, R., Risoleo, M. C., Messina, G., Parisi, L., Carotenuto, M., Vetri, L., & Roccella, M. (2020). The Neurochemistry of Autism. *Brain Sci*, *10*(3), doi:10.3390/brainsci10030163. 10.3390/brainsci10030163 PMC7139720 32182969

[CR43] Matsuo K, Yabuki Y, Fukunaga K (2020). 5-aminolevulinic acid inhibits oxidative stress and ameliorates autistic-like behaviors in prenatal valproic acid-exposed rats. Neuropharmacology.

[CR44] McDougle C, Naylor S, Goodman W, Volkmar F, Cohen D, Price L (1993). Acute tryptophan depletion in autistic disorder: a controlled case study. Biological psychiatry.

[CR45] McDougle CJ, Naylor ST, Cohen DJ, Aghajanian GK, Heninger GR, Price LH (1996). Effects of tryptophan depletion in drug-free adults with autistic disorder. Archives Of General Psychiatry.

[CR46] McLeod R, Mack D, Brown C (1991). Toxoplasma gondii–new advances in cellular and molecular biology. Experimental Parasitology.

[CR47] Melendez JA, Melathe RP, Rodriguez AM, Mazurkiewicz JE, Davies KJ (1999). Nitric oxide enhances the manganese superoxide dismutase-dependent suppression of proliferation in HT-1080 fibrosarcoma cells. Cell Growth & Differentiation.

[CR48] Muller CL, Anacker AMJ, Veenstra-VanderWeele J (2016). The serotonin system in autism spectrum disorder: from biomarker to animal models. Neuroscience.

[CR49] Naushad SM, Jain JM, Prasad CK, Naik U, Akella RR (2013). Autistic children exhibit distinct plasma amino acid profile. Indian Journal Of Biochemistry & Biophysics.

[CR50] Ormstad H, Bryn V, Verkerk R, Skjeldal OH, Halvorsen B, Saugstad OD, Maes M (2018). Serum tryptophan, Tryptophan Catabolites and Brain-derived neurotrophic factor in subgroups of youngsters with Autism Spectrum Disorders. CNS Neurol Disord Drug Targets.

[CR51] Owens DF, Kriegstein AR (2002). Is there more to GABA than synaptic inhibition?. Nature Reviews Neuroscience.

[CR52] Peng MZ, Cai YN, Shao YX, Zhao L, Jiang MY, Lin YT, Liu L (2019). Simultaneous quantification of 48 plasma amino acids by liquid chromatography-tandem mass spectrometry to investigate urea cycle disorders. Clinica Chimica Acta.

[CR53] Proenza AM, Crespí C, Roca P, Palou A (2001). Gender related differences in the effect of aging on blood amino acid compartmentation*. Journal Of Nutritional Biochemistry.

[CR54] Rossignol DA, Frye RE (2012). Mitochondrial dysfunction in autism spectrum disorders: a systematic review and meta-analysis. Molecular Psychiatry.

[CR55] Schafer FQ, Buettner GR (2001). Redox environment of the cell as viewed through the redox state of the glutathione disulfide/glutathione couple. Free Radical Biology And Medicine.

[CR56] Shimmura C, Suda S, Tsuchiya KJ, Hashimoto K, Ohno K, Matsuzaki H, Mori N (2011). Alteration of plasma glutamate and glutamine levels in children with high-functioning autism. PLoS One.

[CR57] Stone TW, Darlington LG (2002). Endogenous kynurenines as targets for drug discovery and development. Nature Reviews. Drug Discovery.

[CR58] Tanianskii, D. A., Jarzebska, N., Birkenfeld, A. L., O’Sullivan, J. F., & Rodionov, R. N. (2019). Beta-aminoisobutyric acid as a Novel Regulator of Carbohydrate and lipid metabolism. *Nutrients*, *11*(3), doi:10.3390/nu11030524. 10.3390/nu11030524 PMC6470580 30823446

[CR59] Van der Leek AP, Yanishevsky Y, Kozyrskyj AL (2017). The Kynurenine Pathway as a Novel link between Allergy and the gut Microbiome. Frontiers In Immunology.

[CR60] Vargason T, Kruger U, McGuinness DL, Adams JB, Geis E, Gehn E, Hahn J (2018). Investigating plasma amino acids for differentiating individuals with Autism Spectrum Disorder and typically developing peers. Res Autism Spectr Disord.

[CR61] Wang S, Zhang S, Liou LC, Ren Q, Zhang Z, Caldwell GA, Witt SN (2014). Phosphatidylethanolamine deficiency disrupts α-synuclein homeostasis in yeast and worm models of Parkinson disease. Proc Natl Acad Sci U S A.

[CR62] Xu G, Strathearn L, Liu B, O’Brien M, Kopelman TG, Zhu J, Bao W (2019). Prevalence and treatment patterns of Autism Spectrum Disorder in the United States, 2016. JAMA Pediatr.

[CR63] Yu X, Qian-Qian L, Cong Y, Xiao-Bing Z, Hong-Zhu D (2021). Reduction of essential amino acid levels and sex-specific alterations in serum amino acid concentration profiles in children with autism spectrum disorder. Psychiatry Research.

[CR64] Yüksel C, Öngür D (2010). Magnetic resonance spectroscopy studies of glutamate-related abnormalities in mood disorders. Biological Psychiatry.

[CR65] Zheng HF, Wang WQ, Li XM, Rauw G, Baker GB (2017). Body fluid levels of neuroactive amino acids in autism spectrum disorders: a review of the literature. Amino Acids.

[CR66] Zhou H, Xu X, Yan W, Zou X, Wu L, Luo X, Team LNS (2020). Prevalence of Autism Spectrum Disorder in China: a Nationwide Multi-center Population-based study among children aged 6 to 12 years. Neuroscience Bulletin.

[CR67] Zou M, Li D, Wang L, Li L, Xie S, Liu Y, Wu L (2020). Identification of amino acid dysregulation as a potential biomarker for Autism Spectrum Disorder in China. Neurotoxicity Research.

[CR68] Zuo, Q. H. (2016). *Social Adaptation Scale for Infants-Junior Middle School Students*. Beijing: HuaXia Press.

